# Analysis of Sociodemographic and Clinical Characteristics of Inflammatory Bowel Disease in Catalonia Based on SIDIAP

**DOI:** 10.3390/jcm13216476

**Published:** 2024-10-29

**Authors:** Cristina García-Serrano, Gloria Mirada, Pepi Estany, Joaquim Sol, Marta Ortega-Bravo, Eva Artigues-Barberà

**Affiliations:** 1 Catalan Health Institute (ICS), Primary Care, 25007 Lleida, Spain; cgarcias.lleida.ics@gencat.cat (C.G.-S.); pestany.lleida.ics@gencat.cat (P.E.); mortega.lleida.ics@gencat.cat (M.O.-B.); eartigues.lleida.ics@gencat.cat (E.A.-B.); 2 Multidisciplinary Research Group on Therapeutics and Interventions in Primary Care (RETICAP Group), Fundació Institut Universitari per a la Recerca a l’Atenció Primària de Salut Jordi Gol i Gurina (IDIAPJGol), Gran Via de les Corts Catalanes, 587, 08007 Barcelona, Spain; 3 Faculty of Nursing and Physiotherapy, University of Lleida, 25198 Lleida, Spain; gloria.mirada@gencat.cat; 4 Public Health Agency of Catalonia, 08005 Lleida, Spain; 5 Lleida Research Support Unit (USR), Fundació Institut Universitari d’Investigació per a la Recerca a l’Atenció Primària de Salut Jordi Gol i Gurina (IDIAPJGol), Rambla Ferran, 44, 25007 Lleida, Spain; 6 Department of Experimental Medicine, University of Lleida-Lleida Biomedical Research Institute (UdL-IRBLleida), 25198 Lleida, Spain; 7 Faculty of Medicine, University of Lleida, Pl. de Víctor Siurana, 1, 25003 Lleida, Spain

**Keywords:** comorbidity, Crohn’s disease, epidemiology, infections, inflammatory bowel disease, SIDIAP, ulcerative colitis

## Abstract

**Background/Objectives:** The increasing global prevalence of inflammatory bowel disease (IBD) presents significant challenges to healthcare systems. Our objective was to identify the sociodemographic and clinical characteristics of IBD patients in Catalonia. **Methods:** A cross-sectional analytical study was carried out on patients diagnosed with IBD in Catalonia (2021). The database of the Information System for the Development of Research in Primary Care of Catalonia was used. **Results:** In Catalonia, the prevalence of IBD was 474 cases per 100,000 people (pcm), with an average diagnosis age of 42.9 years. Crohn’s disease (CD) represented 34.34% of cases, and 21.2% were smokers and 1% were alcoholics. Nutritional status showed 3% underweight, 36.2% overweight, and 20% obese, with only 0.27% diagnosed as malnutrition. Mental health issues are notable; 36,531 pcm patients were diagnosed with anxiety and 14,656 pcm with depression, and 8.24% had a high risk of mortality measured by the Charlson index. The most prevalent vaccine-preventable infections were influenza (19,356 pcm), herpes zoster (8099 pcm), and varicella zoster (6946 pcm), with 4.56% of patients requiring hospitalisation for one of these reasons and 32.8% of patients for IBD complications, with higher rates observed in cases of CD. **Conclusions:** The prevalence of IBD was high, especially in urban areas, and patients showed a relevant number of comorbidities. IBD requires a comprehensive evaluation and interdisciplinary management to improve disease control.

## 1. Introduction

The term “inflammatory bowel disease” (IBD) encompasses a collection of immune-mediated, chronic inflammatory bowel pathologies that affect the gastrointestinal tract and have an unclear aetiology. IBD includes ulcerative colitis (UC), Crohn’s disease (CD), and indeterminate colitis [1].

Onset normally occurs at an early age and includes a chronic course with alternating periods of activity of varying severity [1,2]. Triggers for relapses could be precipitated by certain factors such as nutrition, viral exposures [1,3], smoking [1,4], psychological stress, and other factors modifying the pathogenesis of IBD [1,3]. It can cause digestive complications and extraintestinal manifestations (articular, dermatological, ocular, hepatobiliary, etc.) [1,5]. In addition, it can have an impact on other areas such as fertility and pregnancy, school and work performance, disability, and psychological disorders [6]. For these reasons, IBD generates a high health cost, whether direct (health care, surgical interventions, and/or hospitalisations), indirect (loss of productivity), or intangible (social stigma and loss of quality of life) [2,6,7].

IBD is a pathology that requires follow-up aimed at reducing relapse rate, monitoring side effects, and preventing complications [8]. Current treatment includes the use of drugs such as glucocorticoids, immunosuppressants (azathioprine, methotrexate, mercaptopurine, etc.), and biological therapies (infliximab, adalimumab, vedolizumab, etc.) [1,8,9].

In a systematic review, it was observed that the prevalence of IBD is increasing in North America, in many European countries, in Australia, and in New Zealand [10]. In 2023, the prevalence of IBD was 0.7% in the USA [11] and 0.8% in Canada [12]. In Europe, it is estimated that around 2.5 to 3 million people have been diagnosed with IBD [7,13]. Following this line, several studies have been carried out, such as the ECCO-EpiCom study [14] in Europe and the EpidemIBD study [15] in Spain to describe the incidence and epidemiological and clinical characteristics of IBD. However, researchers claim that nationwide epidemiological studies are necessary to ensure representativeness and limit the impact of regional variability on the results [15].

In this context, real-world data can provide information on a large number of individuals that would allow maximising this representativeness. In the specific case of Catalonia (Spain), the Information System for the Development of Primary Care Research (SIDIAP) database includes high-quality clinical data from 75% of the Catalan population, corresponding to almost 6 million people [16], which would allow a detailed characterisation of practically the entire population of the territory to be obtained.

On the other hand, intestinal diseases in newly industrialised countries are low, but given the increase in incidence in these countries and the increase in prevalence in the aforementioned countries, a significant global burden of this disease is expected. This will bring major challenges for healthcare systems worldwide [10].

The difference in the prevalence and incidence of IBD between countries is due to its multifactorial pathogenesis. Regions with better health standards may have higher rates of IBD, suggesting that lack of early microbial exposure may predispose to autoimmune diseases [17,18]. Likewise, urban areas with greater pollution tend to report more cases of IBD, evidencing a link between environmental factors and gastrointestinal inflammation [17,18]. Conversely, Western diets rich in sugars and fats are associated with a higher incidence of IBD, while Mediterranean diets may offer protection [18]. For this reason, understanding the true prevalence of IBD and characterising this population can help advance our knowledge of how multifactorial causes contribute to the pathogenesis of IBD in different populations.

The aim of this study was to determine the prevalence and sociodemographic and clinical characteristics of subjects diagnosed with IBD in the health districts of Catalonia in 2021.

## 2. Materials and Methods

### 2.1. Type of Study

The current state of the diagnosed population in Catalonia was characterised through a cross-sectional analytical study dated 30 June 2021.

### 2.2. Data Collection

The data were collected from the database of the SIDIAP database. The database includes electronic health records since 2005 from 328 primary care centres managed by the Catalan Health Institute (ICS) in Catalonia (Spain) [16]. This includes data from 8,036,948 individuals, who are automatically included in SIDIAP at the moment they are assigned to a primary care centre of the ICS. This information is recorded in clinical health centres on a daily basis as a result of the clinical practice. The extraction is carried out automatically, without the need for patient recruitment. SIDIAP is a pseudo-anonymised database that does not require informed consent as it does not contain individual personal data [16].

### 2.3. Population

The reference population is composed of living people assigned to a primary care unit in the health districts of Catalonia.

The study population consists of patients who meet the inclusion criteria and do not meet the exclusion criteria.

Inclusion criteria:

- Clinical diagnosis of IBD, including CD (International Classification of Diseases Version 10 (ICD-10: K50*)) and UC (ICD-10: K51*).
- Age equal to or greater than 18 years old at the time of diagnosis.

Exclusion criteria:

- People who had been transferred out of the database before 30 June 2021.
- Deceased before 30 June 2021.

**Figure 1:**
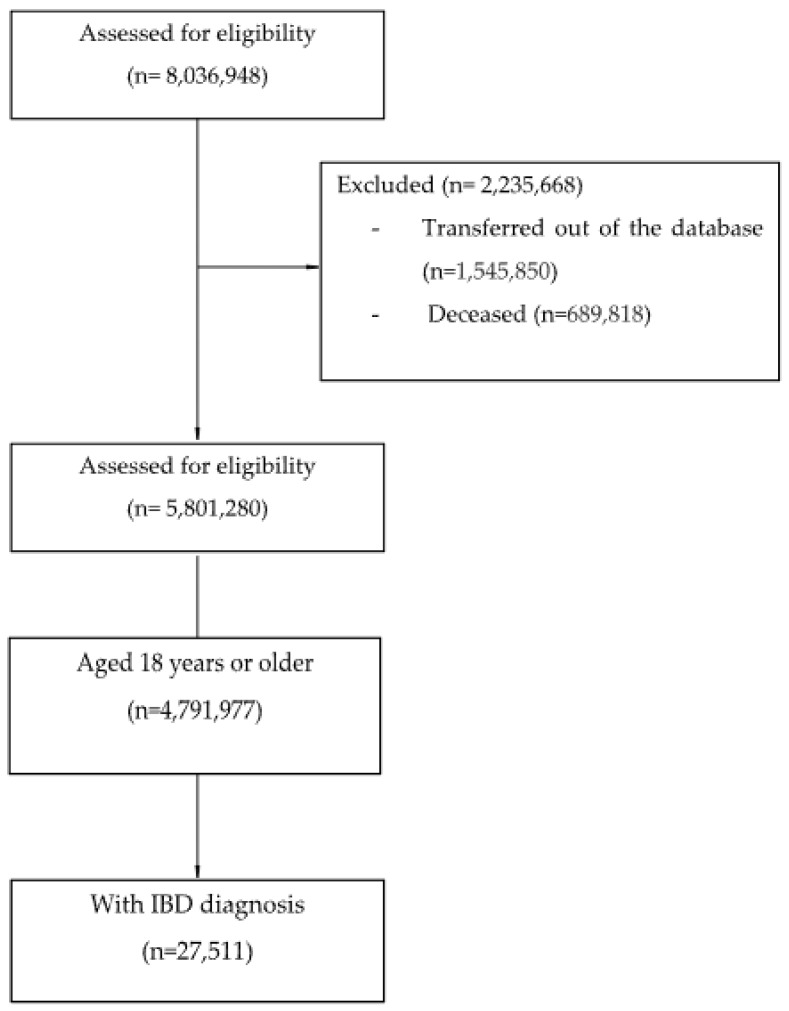
Flowchart of the participant recruitment process.

The recruitment process is shown in Figure 1.

### 2.4. Sampling Procedure

No sample procedure was performed in this study. The final sample for the analysis consisted of all patients who met the stated inclusion criteria and did not meet the exclusion criteria.

### 2.5. Variables

The variables analyzed were sex, age, area of residence (urban/rural), diagnosis (CD/CU), smoking (non-smoker/smoker/ex-smoker), alcohol consumption (non-drinker, low-risk drinker, risk drinker), body mass index (BMI), comorbidities (diabetes mellitus, arterial hypertension, dyslipidaemia, chronic obstructive pulmonary disease, heart failure, liver disease, chronic renal failure, malnutrition, immunodeficiency, cervical dysplasia, malignant and benign neoplasms except benign of skin), immunopreventable infectious diseases (measles, rubella, mumps, chickenpox, herpes zoster, hepatitis A, hepatitis B, hepatitis C, unspecified hepatitis, influenza, pneumonia, tetanus, human papillomavirus), immune-mediated inflammatory diseases (sarcoidosis, amyotrophic lateral sclerosis, multiple sclerosis, psoriasis, hidradenitis suppurativa, rheumatoid arthritis, systemic lupus erythematosus, ankylosing spondylitis, uveitis), mental health disorders (anxiety, depression), Charlson comorbidity index (evaluates life expectancy at 10 years and relates long-term mortality to comorbidity [19]), current immunosuppressive treatment and type of immunosuppressive treatment (Appendix A), number of visits to primary care medicine and nursing, and referrals to other services (gastroenterology, general and digestive surgery, rheumatology, psychiatry, psychology and endocrinology), and number of hospital admissions for immunopreventable infections and due to disease exacerbation. The list of the diagnoses included in each category and their codes from the International Classification of Diseases, Tenth Revision (ICD-10) can be found in Supplementary Table S1.

### 2.6. Statistical Data Analysis

A descriptive analysis was performed. Normality of continuous variables was assessed using a Shapiro–Wilk test and was described using means and standard deviations. Categorical variables were described using absolute and relative frequencies. The prevalence of diseases was expressed as a rate per 100,000 individuals, or per cent mille (pcm). CD and UC groups were compared using Student’s *T* tests, Mann–Whitney U tests, or Chi-squared tests depending on the type and distribution of the variables. Statistical significance was set at *p* < 0.05. The analysis was performed with R version 4.3.2.

### 2.7. Ethics

The study was conducted in accordance with the guidelines of the Declaration of Helsinki and was approved by the Clinical Research Ethics Committee of the Fundació Institut Universitari d’Investigació per a la recerca a l’Atenció Primària de Salut Jordi Gol i Gurina (IDIAPJGol) (code P19/212-P, approved on 20 November 2019). The variables collected were treated anonymously, and the confidentiality of the data is guaranteed under Regulation (EU) 2016/679 of the European Parliament and the Council on Data Protection and applicable national regulations (27 April 2016).

## 3. Results

In the health districts of Catalonia, 27,511 patients have been diagnosed with IBD, which represents a prevalence of 474 pcm. Of the total population with IBD, CD included 9447 patients (34.34%) and UC 18,064 (65.66%). Among those diagnosed, 50.5% were men, with a higher proportion of men in UC and women in CD. The mean age of diagnosis was 42.9 years, being younger in the case of CD, with a mean of 38.4 years, compared to UC, which had a mean age of 45.3 years. The mean age of the IBD population at the time of the cross-section was 54.3 years (Table 1).

When considering the area of residence, prevalence of IBD of 470 pcm and 456 pcm was found in urban and rural areas, respectively. Specifically, 308 pcm and 162 pcm of the urban population had UC and CD, and 320 pcm and 136 pcm of the rural population had UC and CD, respectively. Statistically significant differences were found only in CD prevalence by area (Table 1).

In relation to unhealthy habits, smoking and alcoholism were taken into account. It was observed that 16% of patients with UC were active smokers and 34.40% were ex-smokers. On the other hand, in CD, the observed smoking habit was higher; 31.20% were smokers and 28.60% were ex-smokers. On the other hand, the majority of IBD patients do not consume alcohol, 38.60% have low-risk consumption, and 1% are considered heavy drinkers.

Regarding nutritional level, a prevalence of malnutrition diagnosis of 0.27% was observed. Only 38.90% for UC and 44.80% for CD were in the normal weight range. In UC it was observed that 329 people (2.11%) and in CD 382 people (4.69%) were underweight measured by BMI. Conversely, the figures for overweight (36.20%) and obesity (18.70%) are exceeded in the case of UC, also, with significant differences (Table 2).

In our study, the following comorbidities were notable: arterial hypertension (26,339 pcm), dyslipidaemia (11,054 pcm), diabetes mellitus (10,276 pcm) chronic renal failure (5663 pcm), chronic obstructive pulmonary disease (4155 pcm), and heart failure (1937 pcm) (Table 3). All these conditions were found to be more prevalent in UC, with significant differences (Table 3). However, a higher percentage of patients with CD reported suffering from other immune-mediated inflammatory diseases (12,156 pcm in CD vs. 8863 pcm in UC) (Table 3).

In relation to mental problems, it is observed that 36,531 pcm IBD patients were diagnosed with anxiety, which was more prevalent in CD than in UC. On the other hand, 14,656 pcm patients were diagnosed with depression (Table 3).

Additionally, it was observed that 1701 patients (9.42%) in UC and 567 patients (6%) in CD had a high comorbidity on the Charlson index, which translates into a high risk of mortality at 10 years (Table 3).

Our study shows that 39.30% of the patients visited the emergency department because of IBD, with 32.80% of all patients requiring hospitalisation, and 0.52% being admitted to the intensive care unit. In all three cases, CD patients had higher figures than UC patients, and almost half of CD patients were hospitalised at some point after diagnosis. Regarding the gastroenterology service, 24.90% of the patients were referred to this service from primary care, and 1.26% were referred to the digestive surgery service. In this case, a higher proportion of referrals was observed in UC patients (Table 4).

Regarding hospitalisations due to vaccine-preventable infections, we found that 4.83% of the patients had visited at the emergency department. Additionally, 4.56% of patients required hospital admission, and 0.10% were admitted to the intensive care unit for this reason (Table 4).

Concerning pharmacological treatment, 15.81% of patients with UC and 36.87% of patients with CD were prescribed immunomodulatory therapy, highlighting “other immunosuppressants” that affected 9.29% of patients with UC and 30.50% of patients with CD. Regarding patients with more than one prescribed pharmacological group, we found a higher prevalence in CD than in UC (Table 5).

The most prevalent infections in IBD patients were influenza, herpes zoster, and chickenpox. All infections were more prevalent in CD except hepatitis, which was more prevalent in UC, and 7779 pcm of the patients suffered from pneumonia as a complication of infectious diseases, with the percentage being higher in UC with a significant relationship (Table 6).

Conversely, in our population, we observed a prevalence of human papillomavirus infection of 712 pcm (Table 6), and a prevalence of cervical dysplasia in women of 3104 pcm, which was higher in CD than in UC (Table 3). We also noted a prevalence of genital and anal neoplasms of 603 pcm (Table 3).

## 4. Discussion

### 4.1. Sociodemographic Characteristics

Prevalence rates are variable in countries around the world. In the case of North America and Canada, prevalences of 700 and 800 ppm have been observed, respectively [11,12]. In Western Europe, prevalence rates range from 28.2 to 322/100,000 person-years in CD and from 43.1 to 412/100,000 person-years in patients with UC [10]. However, in southern Europe, lower figures are shown, with a variability of 4.5 to 137.17 in CD and from 14.5 to 133.9 in UC [10].

Although southern European countries, such as Spain, have been considered areas of low prevalence of IBD, our study shows a prevalence of IBD of 474 pcm, much higher than the studies presented. Regarding the sociodemographic variables, our results align with those reported in the Spanish EpidemIBD study, which indicated a mean age of 42 years with approximately 50% men [15]. The prevalence data are consistent with those reported in another study, reinforcing the representativeness and validity of our results [10].

Furthermore, disease rates vary not only between countries but within each country. In relation to Spain, variations were also observed between the estimated prevalence between the different autonomous communities [13]. In Catalonia, a prevalence of 0.59% for CD and 0.34% for UC was estimated [13]. This suggests that many factors influence the pathogenesis of the disease, such as diet, sun exposure, atmospheric pollution, unhealthy habits, and environmental hygiene, among others.

Several studies, including a meta-analysis, have demonstrated that living in an urban area is positively associated with the development of IBD [17,18]. These differences in prevalence in urban areas are more pronounced in CD [20], as seen in our study. Vedamurthy et al. proposed that one hypothesis for the difference in IBD incidence between urban and rural areas is that greater environmental hygiene in urban settings may reduce protective influences, such as exposure to animals and pets, which could contribute to an immune response biased toward autoimmunity and atopy [18].

### 4.2. Unhealthy Habits

Smoking is an environmental and modifiable factor in the risk of IBD since it modulates immune responses and the microbiome, predisposing individuals to intestinal inflammation [18]. Additionally, it has been associated with a higher prevalence of extraintestinal manifestations, such as joint and skin conditions [21]. In the specific case of CD, smoking is not only a risk factor for disease development but has also been identified as the only modifiable risk factor for postoperative recurrence [22].

If we compare the general population with the population with UC in our study, lower numbers of smokers are observed in the case of patients with UC [23]. Conversely, the prevalence of active smokers in CD was twice that of UC and higher than the general Spanish population, representing one-third of the sample. In both UC and CD, approximately one-third of participants were ex-smokers, exceeding the national prevalence of ex-smokers [23]. This indicates some success in smoking cessation efforts among healthcare professionals, but observed figures indicate that further action is needed to enhance these outcomes.

Alcohol has not been definitively linked as a risk factor for the development of IBD or its complications [24]. However, surveys have variably suggested a subjective worsening of gastrointestinal symptoms [24,25], without identifying the specific amount of alcohol that has the greatest impact on these symptoms [25]. Additionally, some research has shown that patients with IBD, as well as those with alcohol use disorders (1% of our population), suffer from intestinal bacterial dysbiosis [25,26], which could entail a greater risk of infections.

In our population, the majority of patients with IBD were abstainers, exceeding the figures for the general population according to the 2020 European Health Survey in Spain [23]. In the case of IBD, it has been observed that between 30% and 60% of patients voluntarily avoid alcohol consumption [24]. These differences may be attributed to education in healthy lifestyle habits or the subjective worsening of symptoms linked to alcohol consumption, as reflected in surveys.

### 4.3. Nutritional Status

Malnutrition is one of the most important factors associated with a poor prognosis in this pathology [27]. This is due to reduced food intake, nutrient malabsorption, loss of enteric nutrients, increased energy requirements due to inflammation, and iatrogenic factors [27]. Proper assessment and treatment of nutritional issues can lead to better symptom control, deeper remission, and an improvement in the quality of life for these patients [28].

The prevalence of protein-energy and specific nutrient malnutrition is higher in CD than in UC, likely because CD can affect any part of the gastrointestinal tract, particularly the small intestine [27]. This is supported in our study, which shows a higher percentage of underweight individuals in CD compared to UC. However, these figures may be underestimated if we consider not only BMI but also other nutritional parameters. Another study found that 10% of IBD patients were underweight as measured by BMI. However, over 30% were malnourished as measured by the Malnutrition Universal Screening Tool (MUST), which accounts for anthropometric measurements, unintentional weight loss, and the impact of acute illness [29]. In our study, a prevalence of malnutrition diagnosis of 0.27% was observed, being higher in CD probably due to greater nutrient malabsorption. This low prevalence compared with the data obtained from BMI may mean under-recording or under-estimation of the problem.

Conversely, obesity has been linked to increased inflammation of the gastrointestinal tract [30]. We observed a higher prevalence of overweight and obesity in UC than in CD, as seen in other studies [31,32]. This could be due to the greater involvement of the digestive tract in CD, leading to consequent weight loss. In the case of CD, we found figures of overweight and obesity similar to those reported by Pringle et al. [30] but much lower than those of other studies [31,32].

The differences between our results based solely on BMI and the estimated prevalence of malnutrition using other screening tools such as MUST highlight the need to carry out screening with validated instruments that consider other nutritional factors beyond anthropometric parameters.

### 4.4. Comorbidities

IBD has been associated with a predisposition to the development of multiple diseases that complicate the condition, leading to a higher risk of complications and mortality. Therefore, it is essential to consider the detection of other comorbidities in this group of patients [6,33]. Bernstein et al. confirm that patients with IBD have greater comorbidity prior to the diagnosis of the disease compared to controls, and this comorbidity increases after diagnosis. This study shows higher prevalences in UC before diagnosis [33], similar to our study, except for immune-mediated diseases, which were more frequent in CD. This could be because UC usually presents at older ages than CD at the time of diagnosis, as observed in our study. However, after diagnosis, an increase in comorbidity was noted in CD [33], which implies that CD is a systemic disease, thereby justifying the greater prevalence of other immune-mediated diseases.

The risks of stroke, ischaemic heart disease, mesenteric ischaemia, atrial fibrillation, and heart failure are increased in patients with IBD [6], particularly among females [34]. Systemic inflammation is known to predispose individuals to premature atherosclerosis [33], which could also be aggravated by the main comorbidities observed in our study, arterial hypertension, dyslipidaemia, and diabetes mellitus. However, cardiovascular mortality has not been shown to increase in this patient population [6,34].

Regarding respiratory manifestations, involvement of the large airways is more common [34]. There is indeed an increased risk of bronchiectasis, interstitial lung disease, and granulomatous lung disease in these patients [6]. The latter has been associated with both infections and medications, including salicylates, methotrexate, thiopurines, and anti-TNF agents [34]. Additionally, IBD-associated interstitial pneumonia has been described [34].

In terms of nephro-urological manifestations, the prevalence of renal failure has been reported to be 2% in CD and up to 15% in hospitalised IBD patients [34]. In our study, a higher prevalence was observed, being higher in UC.

Finally, the impact of comorbidity in our patients was measured using the Charlson index, observing a high risk of mortality at 10, especially in UC. Despite CD being described as a more serious pathology, it is observed that patients with UC showed a more comorbid profile and a higher risk of mortality associated with their comorbidity. This could be due to the higher prevalence of comorbidities in UC and also to the older age of these patients.

### 4.5. Anxiety and Depression

People with IBD frequently experience psychological disorders, whether or not they are directly associated with the disease, with anxiety and depression being particularly prominent. There are several pathways through which stress can exert this effect, including inhibition of the vagus nerve, production of pro-inflammatory cytokines, modification of the gut microbiome, and increased intestinal permeability [20]. Additionally, an effect of stress and depression on the frequency and intensity of flare-ups has been described [35], suggesting that psychological disorders can modulate the clinical expression of IBD [8,35].

In Spain, the most prevalent mental disorders in the general population (2020) are anxiety, with a prevalence of 6.7%, and depression, with a prevalence of 4.1%. In relation to patients with IBD, a systematic review indicates a prevalence of anxiety of 20% and a prevalence of depression of 15% [36]. In our study, one in three patients with IBD was diagnosed with anxiety, being more prevalent in CD than in UC and 5.45 times more prevalent than in the general population. On the other hand, one in seven patients was diagnosed with depression (3.58 times more than in the general population).

Given the prevalence of these disorders in IBD, it is essential to detect, assess, and monitor them. The need for psychiatric referral should also be considered, especially given the risk of suicidal ideation that has been observed in patients with severe depression [37]. In our study, we found that one in ten patients had been referred to psychology and/or psychiatry, which is a low proportion compared with anxiety and depression diagnoses. Notably, the proportion of referred patients was higher in CD. This may indicate that, in Catalonia, the diagnosis and monitoring of mental disorders are primarily being handled by primary care.

Furthermore, stress, anxiety, and depressive symptoms have been identified as significant predictors of health-related quality of life [38]. Therefore, psychological interventions aimed at addressing these potentially modifiable factors should be considered [38]. According to ECCO guidelines, nurses should identify the patient’s needs and develop an active and empathetic listening approach, promoting comprehensive support [6].

### 4.6. Treatment

According to the British and ECCO guidelines [39], oral aminosalicylates (5-ASA) are the standard therapy for UC. In the case of CD, initiation with 5-ASA is not recommended; glucocorticoids should be used instead. In addition, early introduction of thiopurines (azathioprine or mercaptopurine) or methotrexate [9] is advised. Additionally, some patients should be considered for biological therapy [9,40].

It has been observed that not all treatments for IBD cause immunosuppression, and those that do can produce varying degrees of it. While 5-ASA is not associated with immunosuppression, systemic corticosteroids, thiopurines, and anti-TNF agents are linked to an increased risk of opportunistic infections. Studies, including a meta-analysis, suggest that combination therapies carry a higher risk [41,42], with the odds ratio increasing from 2.9 for one immunosuppressive drug to 14.5 for two or three [41].

In our study, the most prescribed drugs were “other immunosuppressants”, with higher usage in CD than in UC, in accordance with protocol recommendations. Glucocorticoids were the next most prescribed drugs, with no significant differences between the two pathologies. This suggests that both types of IBD share a similar need for treatment with glucocorticoids, which are used to reduce inflammation and control relapses. Regarding patients with more than one prescribed pharmacological group, we found a higher prevalence in CD than in UC. CD can affect different parts of the gastrointestinal tract, which may require a more diverse approach to treatment, including multiple classes of medications (immunosuppressants, biologics, etc.) to control inflammation and symptoms, and prevent complications.

### 4.7. Most Common Infections in IBD

Opportunistic infections are complications derived from immunosuppression caused by the disease itself and by the prescribed treatments. One study reports that 19% of relevant infections in patients with IBD were opportunistic and mostly occurred after the start of immunosuppressants and/or biological therapies [43]. In our study, the most prevalent infections were influenza, herpes zoster, and varicella zoster.

A study of 140,480 patients showed that IBD carried a higher risk of influenza compared to the general population and, in addition, they were more likely to require hospitalisation [44]. In fact, in our study, it was observed that influenza was the most frequent infection, with a prevalence of 19,356 pcm. Furthermore, at the respiratory level, it was observed that 7779 pcm of the patients presented pneumonia, with higher figures in the case of the UC. One study stated that IBD patients are at higher risk of having bacterial and viral pneumonia [45]. For this reason, pneumococcal and influenza vaccinations are recommended in these patients.

Herpes zoster is one of the most frequent opportunistic infections observed in immunosuppressed IBD patients and is particularly associated with thiopurines and tofacitinib [41]. In the study by Zabana et al., a prevalence of herpes zoster of 3.9/1000 was observed [43]. In our study, this infection was also observed to be one of the most frequent infections, with a prevalence of 8099 pcm. Recently, in Spain, herpes zoster vaccination has been indicated for people aged 50 years and older undergoing treatment with immunomodulatory or immunosuppressive drugs [46].

Furthermore, the immunosuppression inherent to IBD increases the risk of developing reactivation of viruses such as hepatitis B and varicella zoster, and this risk, in turn, increases with age [41]. It is possible that age is one of the reasons why hepatitis was more common in UC in our study.

Conversely, the human papillomavirus showed a prevalence of 712 pcm in our study. Furthermore, cervical dysplasia was diagnosed in 3104 pcm of the population, with a higher prevalence in patients with CD compared to those with UC. Genital and anal neoplasms were observed in 603 pcm of the patients. These findings suggest a possible association between CD and an increased risk of human papillomavirus complications and cervical dysplasia.

In conclusion, the most prevalent infectious diseases in IBD are vaccine-preventable, highlighting the importance of recruiting patients by risk groups to implement this preventive measure [47,48]. In the case of human papillomavirus infection, it is also essential to incorporate early detection of cancer associated with this infection. In fact, it is recommended that all women with IBD receiving systemic immunosuppressive therapy undergo an annual cervical cancer screening test [9,49,50].

### 4.8. Healthcare Management

The goal of IBD treatment is to induce and maintain remission and improve health-related quality of life [6]. This requires early diagnosis, strict control of disease activity, close monitoring to avoid treatment side effects, and an interdisciplinary and holistic approach [51]. The interdisciplinary team in the hospital setting, as described in the ECCO quality standards, is composed of at least one gastroenterologist/endoscopist, nurse, radiologist, pathologist, and surgeon dedicated to IBD [51].

The primary care setting is the patient’s first contact with the healthcare system, so the start of diagnostic guidance and follow-up of most of the pathology is carried out in this setting. When there is a suspicion of IBD, patients must be referred to the gastroenterology service to make a definitive diagnosis and establish treatment guidelines [2]. According to our data, most visits are made in primary care by both medical and nursing staff, regardless of the IBD subtype. Primary care physicians and nurses are essential for long-term management, including monitoring disease progression, managing medications, and coordinating care with specialists. This is especially important for IBD, as the condition requires regular follow-ups to evaluate symptoms, adjust treatment plans, and provide preventive care.

Nurses play a key role in the treatment of IBD through education, support, patient counselling, monitoring and administration of therapies, and prevention of complications [47,48]. Preventive measures include, among others, vaccinations, analytical controls, smoking cessation, cancer prevention, control of comorbidities such as hypertension and hypercholesterolaemia, and management of depression [1,6].

The digestive complications of IBD, its extraintestinal manifestations, or the impact of the disease on other areas such as school or work performance or psychological disorders require an interdisciplinary and specialised approach [6]. Regarding emergency department visits, hospitalisations, and intensive care unit admission for IBD, patients with CD had higher numbers than patients with UC, and almost half of patients with CD were hospitalised at some point after diagnosis. This indicates that CD may lead to more severe complications or exacerbations that require more intensive medical intervention.

In Spain, a survey of healthcare professionals was conducted, revealing deficiencies in the relationship between primary care providers and gastroenterology services due to the lack of developed joint protocols and quick and effective means of communication [2]. Effective communication and coordination between primary care and hospital care are indeed crucial for ensuring the best outcomes for patients who require care from multiple health disciplines.

### 4.9. Limitations and Strengths

The strength of our study lies in the representativeness of the sample, as it includes all patients diagnosed with IBD in the health districts of Catalonia. The database used is SIDIAP, which encompasses data from 328 primary care centres managed by the Institut Català de la Salut in Catalonia, Spain. As of June 2021, SIDIAP had 5.8 million active individuals and included 75% of the Catalan population, making it representative of the general population living in Catalonia [16].

However, information biases may arise in our study because the quality of SIDIAP data depends on primary care professionals accurately recording clinical information, which may lead to the underreporting of certain variables.

Another limitation is that indeterminate colitis was not included. Indeterminate colitis is diagnosed in 10% of patients with IBD. This pathology includes patients in whom clinical, endoscopic, and histological distinction between UC and CD of the colon is not possible at the time of diagnosis. However, later on, clear signs of CD or UC may appear. For this reason, we have analysed the two diseases characteristic of IBD.

## 5. Conclusions

The prevalence of IBD in Catalonia was 474 pcm, being higher in ulcerative colitis. These were young adults with an equal distribution between sexes and with a greater frequency of residence in urban areas. Regarding unhealthy habits, a high prevalence of tobacco and alcohol consumption was observed. Furthermore, although most patients remained below or above the ideal body mass index, the diagnosis of malnutrition was low.

Regarding comorbidities, the most frequent are related to emotional well-being in both pathologies. One in three patients presented anxiety and one in seven, depression. Other prevalent diseases observed were arterial hypertension, dyslipidaemia, and diabetes mellitus, especially in UC. In the case of CD, immune-mediated diseases stood out in second place. Despite age, one in twelve patients has a high risk of mortality.

Concerning infections, influenza was the most prevalent in both diseases. The second most common infection in UC was herpes zoster, followed by chickenpox and, vice versa, in the case of CD.

Regarding hospitalisations, almost half of the patients with CD and a quarter of the patients with UC required at least one hospital admission due to a disease outbreak. The average number of hospital admissions for CD and UC was 2.63 and 2.03 times, respectively, in patients who had already been hospitalised. In the case of hospitalisation due to infection, five out of every hundred patients were admitted.

The majority of hospital referrals were made to the gastroenterology service, followed by general surgery. The follow-up of these patients was carried out mostly in primary care.

As future perspectives for the population, we recommend awareness campaigns about unhealthy habits and prevention measures in these patients. In relation to professionals, improvements must be made in adapting screening and vaccination protocols for IBD at the time of diagnosis. In addition, routine mental health assessment and access to appropriate therapies should be included. Telemedicine could play an important role in monitoring these patients, facilitating access to care and allowing continued monitoring of their health.

Continuous research on IBD could lead to the development of new recommendations that not only focus on symptom control but also on improving quality of life and reducing comorbidities. Further research should include forecasting models for predicting the evolution of the disease and prognostic models.

## Figures and Tables

**Table 1:** Sociodemographic characteristics and unhealthy habits in patients diagnosed with IBD in the health districts of Catalonia, (Spain), 2021.

Sociodemographic Characteristics				
	All (*n =* 27,511)	Ulcerative Colitis (*n =* 18,064)	Crohn’s Disease (*n =* 9477)	*p*-Value
**Sex**				<0.001
Women	13,628 (49.50%)	8772 (48.60%)	4856 (51.40%)	
Men	13,883 (50.50%)	9292 (51.40%)	4591 (48.60%)	
**Age of diagnosis**	42.9 (16.3)	45.3 (16.0)	38.4 (15.9)	<0.001
**Age at June 2021**	54.3 (16.7)	56.8 (16.5)	49.4 (16.0)	<0.001
**Residence area**				<0.001
Rural area	1519 (5.94%)	1066 (6.33%)	453 (5.18%)	
Urban area	24,073 (94.10%)	15,778 (93.70%)	8295 (94.80%)	
**Unhealthy habits**				
**Tobacco consumption**				<0.001
Non-smoker	11,919 (46.40%)	8383 (49.60%)	3536 (40.20%)	
Smoker	5448 (21.20%)	2707 (16.00%)	2741 (31.20%)	
Ex-smoker	8327 (32.40%)	5817 (34.40%)	2510 (28.60%)	
**Alcohol consumption**				<0.001
Non-drinker	14,995 (60.40%)	9705 (59.30%)	5290 (62.60%)	
Low risk consumption	9575 (38.60%)	6499 (39.70%)	3076 (36.40%)	
Heavy drinker	249 (1.00%)	169 (1.03%)	80 (0.95%)	

**Table 2:** Nutritional status in patients diagnosed with IBD in the health districts of Catalonia (Spain), 2021.

	All (*n =* 27,511)	Ulcerative Colitis (*n =* 18,064)	Crohn’s Disease (*n =* 9477)	*p*-Value
**BMI (numeric)**	26.2 (5.04)	26.5 (4.89)	25.7 (5.28)	<0.001
**BMI (categorical)**				<0.001
Underweight	711 (2.99%)	329 (2.11%)	382 (4.69%)	
Normal weight	9715 (40.90%)	6071 (38.90%)	3644 (44.80%)	
Obesity	4440 (18.70%)	3048 (19.50%)	1392 (17.10%)	
Morbid obesity	306 (1.29%)	198 (1.27%)	108 (1.33%)	
Overweight	8592 (36.20%)	5976 (38.30%)	2616 (32.10%)	
**Malnutrition**	74 (0.27%)	32 (0.18%)	42 (0.44%)	<0.001

BMI: Body Mass Index.

**Table 3:** Comorbidities in patients diagnosed with IBD in the health districts of Catalonia (Spain), 2021.

Prognostic Comorbidity				
	All (*n =* 27,511)	Ulcerative Colitis (*n =* 18,064)	Crohn’s Disease (*n =* 9477)	*p*-Value
**Charlson index (numeric)**	0.76 (1.23)	0.82 (1.29)	0.65 (1.11)	<0.001
**Charlson index (categorical)**				<0.001
High comorbidity	2268 (8.24%)	1701 (9.42%)	567 (6.00%)	
Absence of comorbidity	15,967 (58.00%)	10,182 (56.40%)	5785 (61.20%)	
Low comorbidity	9276 (33.70%)	6181 (34.20%)	3095 (32.80%)	
**Cardiovascular diseases**				
**Dyslipidaemia**	3401 (11,054 pcm)	2552 (14,128 pcm)	849 (8959 pcm)	<0.001
**Arterial hypertension**	7246 (26,339 pcm)	5245 (29,036 pcm)	2001 (21,114 pcm)	<0.001
**Heart failure**	533 (1937 pcm)	398 (2203 pcm)	135 (1425 pcm)	<0.001
**Endocrinological diseases**				
**Diabetes Mellitus**	2827 (10,276 pcm)	2111 (11,686 pcm)	716 (7555 pcm)	<0.001
**Respiratory diseases**				
**Chronic obstructive pulmonary disease**	1143 (4155 pcm)	816 (4517 pcm)	327 (3450 pcm)	<0.001
**Mental disorders**				
**Anxiety**	10,050 (36,531 pcm)	6353 (35,169 pcm)	3697 (39,010 pcm)	<0.001
**Depression**	4032 (14,656 pcm)	2629 (14,554 pcm)	1403 (14,804 pcm)	0.519
**Cervical dysplasia/neoplasms**				
**Cervical dysplasia**	423 (3104 pcm)	206 (2348 pcm)	217 (4469 pcm)	<0.001
**Neoplasms**	166 (603 pcm)	98 (543 pcm)	68 (718 pcm)	0.085
**Other diseases**				
**Immune-mediated inflammatory diseases**	2753 (10,007)	1601 (8863 pcm)	1152 (12,156 pcm)	<0.001
**Chronic renal failure**	1558 (5663 pcm)	1146 (6344 pcm)	412 (4347 pcm)	<0.001

pcm: per cent mille.

**Table 4:** Visits, referrals, and hospitalisations in patients diagnosed with IBD in the health districts of Catalonia (Spain), 2021.

Referrals and Total Visits				
	All (*n =* 27,511)	Ulcerative Colitis (*n =* 18,064)	Crohn’s Disease (*n =* 9477)	*p*-Value
Any referral to gastroenterology	6849 (24.90%)	4720 (26.10%)	2129 (22.50%)	<0.001
Number of referrals to gastroenterology (in referred patients)	1.45 (0.84)	1.46 (0.85)	1.42 (0.82)	0.026
Any referral to general surgery	3267 (11.90%)	2175 (12.00%)	1092 (11.60%)	0.249
Number of referrals to general surgery (in referred patients)	1.31 (0.67)	1.31 (0.66)	1.32 (0.67)	0.808
Any referral to psychiatry/psychology	2744 (9.97%)	1594 (8.82%)	1150 (12.20%)	<0.001
Number of referrals to psychiatry/psychology (in referred patients)	1.50 (0.92)	1.48 (0.88)	1.54 (0.97)	0.098
Any referral to rheumatology	2448 (8.90%)	1629 (9.02%)	819 (8.67%)	0.346
Number of referrals to rheumatology (in referred patients)	1.38 (0.79)	1.39 (0.81)	1.35 (0.75)	0.227
Any referral to endocrinology	976 (3.55%)	638 (3.53%)	338 (3.58%)	0.872
Number of referrals to endocrinology (in referred patients)	1.27 (0.62)	1.29 (0.62)	1.24 (0.62)	0.243
Any referral to digestive surgery	346 (1.26%)	247 (1.37%)	99 (1.05%)	0.028
Number of referrals to digestive surgery (in referred patients)	1.08 (0.30)	1.09 (0.31)	1.05 (0.26)	0.294
Any visit by general practitioners	27,106 (98.50%)	17,800 (98.50%)	9306 (98.50%)	0.88
Number of visits by general practitioners (in patients visited)	53.3 (49.3)	54.5 (50.6)	51.1 (46.6)	<0.001
Any visit by nurses	25,897 (94.10%)	16,939 (93.80%)	8958 (94.80%)	<0.001
Number of visits per nurse (in patients visited)	28.5 (42.8)	27.8 (41.5)	29.8 (45.2)	<0.001
**Hospitalisations**				
**Due to IBD**				
Any visit to the emergency department	10,806 (39.30%)	5681 (31.40%)	5125 (54.30%)	<0.001
Number of emergency department visits (in visited patients)	2.79 (2.79)	2.35 (2.35)	3.28 (3.14)	<0.001
Any hospitalisation	9018 (32.80%)	4570 (25.30%)	4448 (47.10%)	<0.001
Number of hospitalisations (in hospitalised patients)	2.33 (2.15)	2.03 (1.83)	2.63 (2.39)	<0.001
Any admission to Intensive Care Unit	142 (0.52%)	63 (0.35%)	79 (0.84%)	<0.001
Number of admissions to Intensive Care Unit (in admitted patients)	1.20 (0.50)	1.21 (0.60)	1.20 (0.40)	0.966
**Due to infection**				
Any visit to the emergency department	1330 (4.83%)	830 (4.59%)	500 (5.29%)	0.011
Number of emergency department visits (in visited patients)	1.38 (0.94)	1.37 (0.96)	1.38 (0.89)	0.834
Any hospitalisation	1255 (4.56%)	781 (4.32%)	474 (5.02%)	0.01
Number of hospitalisations (in hospitalised patients)	1.36 (0.93)	1.36 (0.95)	1.37 (0.90)	0.804
Any admission to Intensive Care Unit	27 (0.10%)	15 (0.08%)	12 (0.13%)	0.366
Number of admissions to Intensive Care Unit (in admitted patients)	1.48 (0.85)	1.27 (0.46)	1.75 (1.14)	0.188

**Table 5:** Treatment (active medical prescriptions) in patients diagnosed with IBD in the health districts of Catalonia (Spain), 2021.

	All (*n =* 27,511)	Ulcerative Colitis (*n =* 18,064)	Crohn’s Disease (*n =* 9477)	*p*-Value
**Glucocorticoids**	1525 (5.54%)	1002 (5.55%)	523 (5.54%)	0.992
**Selective immunosuppressants**	47 (0.17%)	36 (0.20%)	11 (0.12%)	0.154
**Calcineurin inhibitors**	206 (0.75%)	139 (0.77%)	67 (0.71%)	0.633
**Other immunosuppressants**	4565 (16.60%)	1679 (9.29%)	2886 (30.50%)	<0.001
**Total prescribed drugs:**				<0.001
0	21,697 (78.90%)	15,491 (85.8%)	6206 (65.70%)	
1	5310 (19.30%)	2307 (12.80%)	3003 (31.80%)	
2	478 (1.74%)	249 (1.38%)	229 (2.42%)	
3	26 (0.09%)	17 (0.09%)	9 (0.10%)	
**Multiple drugs**	504 (1.83%)	266 (1.47%)	238 (2.52%)	<0.001

**Table 6:** Infectious diseases in patients diagnosed with IBD in the health districts of Catalonia (Spain), 2021.

	All (*n =* 27,511)	Ulcerative Colitis (*n =* 18,064)	Crohn’s Disease (*n =* 9477)	*p*-Value
**Other infectious diseases**	273 (992 pcm)	151 (836 pcm)	122 (1287 pcm)	<0.001
**Influenza**	5325 (19,356 pcm)	3400 (18,822 pcm)	1925 (20,312 pcm)	0.002
**Hepatitis A**	80 (291 pcm)	56 (310 pcm)	24 (253 pcm)	0.484
**Hepatitis B**	219 (796 pcm)	157 (869 pcm)	62 (654 pcm)	0.07
**Hepatitis C**	274 (996 pcm)	184 (1019 pcm)	90 (950 pcm)	0.646
**Unspecified hepatitis**	101 (367 pcm)	61 (338 pcm)	40 (422 pcm)	0.312
**Herpes zoster**	2228 (8099 pcm)	1436 (7950 pcm)	792 (8357 pcm)	0.219
**Mumps**	74 (269 pcm)	47 (260 pcm)	27 (285 pcm)	0.789
**Rubella**	36 (131 pcm)	22 (122 pcm)	14 (148 pcm)	0.689
**Tetanus**	0 (0 pcm)	0 (0 pcm)	0 (0 pcm)	-
**Chickenpox**	1911 (6946 pcm)	972 (5381 pcm)	939 (9908 pcm)	<0.001
**Measles**	120 (436 pcm)	69 (382 pcm)	51 (538 pcm)	0.073
**Human papillomavirus**	196 (712 pcm)	109 (603 pcm)	87 (918 pcm)	0.004
**Pneumonia**	2140 (7779 pcm)	1455 (8054 pcm)	685 (7228 pcm)	0.019

pcm: per cent mille.

## Data Availability

In accordance with current European and national law, we are not allowed to distribute or make publicly available the data to other parties. Researchers from public institutions can request data from SIDIAP if they comply with certain requirements. Further information is available online (https://www.sidiap.org/index.php/en/solicituds-en (accessed on 10 September 2024)).

## Supplementary Materials

The following supporting information can be downloaded at: https://www.mdpi.com/article/10.3390/jcm13216476/s1, Table S1: List of diagnosis codes and description.

## Appendix A

**Monoclonal Antibodies**
Rituximab
Ofatumumab
Brentuximab vedotin
Daratumumab
Gemtuzumab ozogamicin
**Immunosuppressants**
Selective immunosuppressants
Mycophenolate mofetil
Sirolimus
Natalizumab
Abatacept
Eculizumab
Belimumab
Fingolimod
Teriflunomide
Apremilast
Alemtuzumab
Everolimus
Leflunomide
Vedolizumab
Belatacept
Ocrelizumab
Tofacitinib
Baricitinib
Muromonab-CD3
Tumour necrosis factor alpha inhibitors (anti-TNF)
Etanercept
Infliximab
Adalimumab
Certolizumab pegol
Golimumab
Interleukin inhibitors
Basiliximab
Anakinra
Ustekinumab
Tocilizumab
Canakinumab
Ixekizumab
Secukinumab
Siltuximab
Calcineurin inhibitors
Ciclosporina
Tacrolimus
Other immunosuppressants
Azathioprine
Lenalidomide
Pirfenidone
Pomalidomide
Methotrexate
Thalidomide
**Systemic glucocorticoids**
Dexamethasone
Methylprednisolone
Prednisone
Deflazacort
Hydrocortisone
Prednisolone
