# Supplementary material for: Analysis of Sociodemographic and Clinical Characteristics of Inflammatory Bowel Disease in Catalonia Based on SIDIAP
Source: J Clin Med. 2024 Oct 29;13(21):6476. doi: 10.3390/jcm13216476 (PMC11545972; doi:10.3390/jcm13216476)
Supplement: Supplementary file 1 [file jcm-13-06476-s001.zip › jcm-3281785-supplementary.pdf]

Supplementary Table S1. List of diagnosis codes and description

Variable codes	
**Variable**	**Code**
Diagnostics (ICD-10)	
Crohn's disease	K50*
Ulcerative colitis	K51*
Comorbilidades (CIE-10)	
Diabetes mellitus	E08* Diabetes mellitus due to underlying condition<br>E09* Drug or chemical induced diabetes mellitus<br>E10* Type 1 diabetes mellitus<br>E11* Type 2 diabetes mellitus
Hypertension	I10* Essential (primary) hypertension<br>I15* Secondary hypertension
Dyslipidaemia	E78.9 Disorder of lipoprotein metabolism, unspecified
Obesity	E66* Overweight and obesity<br>O99.21* Obesity complicating pregnancy, childbirth, and the puerperium
Chronic obstructive pulmonary disease	J44* Other chronic obstructive pulmonary disease
Heart failure	I50* Heart failure<br>I97.11* Postprocedural cardiac insufficiency<br>I97.13* Postprocedural heart failure
Liver diseases	A51.45 Secondary syphilitic hepatitis<br>B15* Acute hepatitis A<br>B16* Acute hepatitis B<br>B17* Other acute viral hepatitis<br>B18* Chronic viral hepatitis<br>B19* Unspecified viral hepatitis<br>B58.1 Toxoplasma hepatitis<br>C22 Malignant neoplasm of liver and intrahepatic bile ducts<br>K70* Alcoholic liver disease<br>K71 Toxic liver disease<br>K73* Chronic hepatitis, not elsewhere classified<br>K75* Other inflammatory liver diseases<br>K76 Other diseases of liver<br>R16 Hepatomegaly and splenomegaly, not elsewhere classified
Chronic kidney disease	I12* Hypertensive chronic kidney disease<br>N18* Chronic kidney disease<br>Q61* Cystic kidney disease
Injury of spleen	S36.0* Injury of spleen
Malnutrition	E43* Unspecified severe protein-calorie malnutrition<br>E44* Protein-calorie malnutrition of moderate and mild degree<br>E46 Unspecified protein-calorie malnutrition<br>R63.6 Underweight
Dysplasia of cervix uteri	N87* Dysplasia of cervix uteri
Neoplasm	C21.0 Malignant neoplasm of anus, unspecified<br>C52 Malignant neoplasm of vagina<br>C53.9 Malignant neoplasm of cervix uteri, unspecified<br>C63 Malignant neoplasm of other and unspecified male genital organs

Variable codes	
**Variable**	**Code**
	D06 Carcinoma in situ of cervix uteri<br>D06.0 Carcinoma in situ of endocervix<br>D06.1 Carcinoma in situ of exocervix<br>D06.7 Carcinoma in situ of other parts of cervix<br>D06.9 Carcinoma in situ of cervix, unspecified<br>D29.0 Benign neoplasm of penis<br>D12.9 Benign neoplasm of anus and anal canal<br>D28.0 Benign neoplasm of vulva<br>D28.1 Benign neoplasm of vagina
Immunodeficiency	B97.3 Retrovirus as the cause of diseases classified elsewhere<br>D80 Immunodeficiency with predominantly antibody defects<br>D81 Combined immunodeficiencies<br>D82 Immunodeficiency associated with other major defects<br>D83 Common variable immunodeficiency<br>D84 Other immunodeficiencies
Infectious diseases	B01* Varicella [chickenpox]<br>B02 Zoster [herpes zoster]<br>B05 Measles<br>B06 Rubella [German measles]<br>B16* Acute hepatitis B<br>B17.0 Acute delta-(super) infection of hepatitis B carrier<br>B18.0 Chronic viral hepatitis B with delta-agent<br>B18.1 Chronic viral hepatitis B without delta-agent<br>B25* Cytomegaloviral disease<br>B26* Mumps<br>B96.0 Mycoplasma pneumoniae [M. pneumoniae] as the cause of diseases classified elsewhere<br>B96.1 Klebsiella pneumoniae [K. pneumoniae] as the cause of diseases classified elsewhere<br>B96.3 Hemophilus influenzae [H. influenzae] as the cause of diseases classified elsewhere<br>B97.7 Papillomavirus as the cause of diseases classified elsewhere<br>G00* Bacterial meningitis, not elsewhere classified<br>J10* Influenza due to other identified influenza virus<br>J11* Influenza due to unidentified influenza virus<br>P35* Congenital viral diseases<br>Z22 Carrier of infectious disease
Pneumonia	A01.03 Typhoid pneumonia<br>B01.2 Varicella pneumonia<br>B06.81 Rubella pneumonia<br>J12* Viral pneumonia, not elsewhere classified<br>J13 Pneumonia due to Streptococcus pneumoniae<br>J14 Pneumonia due to Hemophilus influenzae<br>J15* Bacterial pneumonia, not elsewhere classified<br>J16* Pneumonia due to other infectious organisms, not elsewhere classified<br>J17* Pneumonia in diseases classified elsewhere<br>J18* Pneumonia, unspecified organism
Immune-mediated inflammatory diseases	D86* Sarcoidosis<br>G12.21 Amyotrophic lateral sclerosis<br>G35* Multiple sclerosis<br>L40* Psoriasis<br>L73.2 Hidradenitis suppurativa

Variable codes	
**Variable**	**Code**
	M05* Rheumatoid arthritis with rheumatoid factor<br>M06* Other rheumatoid arthritis<br>M08* Juvenile arthritis<br>M32* Systemic lupus erythematosus (SLE)<br>M45* Ankylosing spondylitis
Depressive disorder	F32* Depressive episode<br>F33* Major depressive disorder, recurrent
Anxiety disorders	F06* Other mental disorders due to known physiological condition<br>F40* Phobic anxiety disorders<br>F41* Other anxiety disorders
Immunosuppressive treatment (ATC: Anatomical, Therapeutic, Chemical classification system)	
Monoclonal antibodies	Rituximab (L01XC02), ofatumumab (L01XC10), brentuximab vedotin (L01XC12), daratumumab (L01XC24), gemtuzumab ozogamicin (L01XC05)
Selective immunosuppressant	Mycophenolate mofetil, sirolimus (L04AA10, S01XA23, L01XE09), natalizumab (L04AA23), abatacept (L04AA24), eculizumab (L04AA25), belimumab (L04AA26), fingolimod (L04AA27), teriflunomide (L04AA31), apremilast (L04AA32), alemtuzumab (L04AA34), everolimus (L01XE10, L04AA18), leflunomide (L04AA13), vedolizumab (L04AA33), belatacept (L04AA28), ocrelizumab (L04AA36), tofacitinib (L04AA29), baricitinib (L04AA37), muromonab-CD3 (L04AA02)
Tumor necrosis factor alpha inhibitors	Etanercept (L04AB01), infliximab (L04AB02), adalimumab (L04AB04), certolizumab pegol (L04AB05), golimumab (L04AB06)
Interleukin inhibitors	Basiliximab (L04AC02), anakinra (L04AC03), ustekinumab (L04AC05), tocilizumab (L04AC07), canakinumab (L04AC08), ixekizumab (L04AC13), secukinumab (L04AC10), siltuximab (L04AC11)
Calcineurin inhibitors	Ciclosporin (L04AD01, S01XA18), tacrolimus (D11AH01, L04AD02)
Other immunosuppressants	Azathioprine (L04AX01), lenalidomide (L04AX04), pirfenidone (L04AX05), pomalidomide (L04AX06), methotrexate (L01BA01, L04AX03), thalidomide (L04AX02).
Systemic glucocorticoids	Dexamethasone (A01AC02, C05AA09, D07AB19, D07XB05, D07CB04, D10AA03, H02AB02, R01AD03, S01BA01, S01CB01, S02BA06, S03BA01, S01CA01, S02CA06, S03CA01, R01AD53), nisolone (D07AA01, D10AA02, H02AB04, D07AC14, D07CA02, S01CA08, H02BX019 , prednisone (S01CA09, H02AB15, A07EA03, H02AB079, deflazacort (H02AB13), hydrocortisone (A01AC03, A07EA02, C05AA01, D07AA02, D07XA01, H02AB09, S01BA02, S01CB03, S02BA01, , D07CA01, S01CA03, S02CA03, S03CA04, D07BA04, S01BB01, D07AB11, D07AB02, D07BB04, R01AD60), prednisolone (D07AA01, D10AA02, H02AB04, D07AC14, D07CA02, S01CA08, H02BX01, A07EA01, C05AA04, D07AA03, D07XA02, H02AB06, AD02, S01BA04, S01CB02, S02BA03, S03BA02, D07CA03, S01CA02, S02CA01 , S03CA02, D07BA01, S01BB02, V03AB05, A01AC54, R01AD52)
